# Prevalence of *Chlamydophila* spp. and Canid herpesvirus-1 in Polish dogs

**DOI:** 10.14202/vetworld.2024.226-232

**Published:** 2024-01-25

**Authors:** Kinga Domrazek, Piotr Jurka

**Affiliations:** Laboratory of Small Animal Reproduction, Institute of Veterinary Medicine, Warsaw University of Life Sciences, Nowoursynowska 159C Street, Warsaw 02-787, Poland

**Keywords:** *Chlamydia*, canid herpes virus 1, dog, herpesvirus, semen

## Abstract

**Background and Aim:**

*Chlamydophila* spp. affect Leydig and Sertoli cells by dysregulating spermatogenesis, inducing apoptosis and sperm DNA fragmentation, as well as benign prostate hyperplasia. Canid herpes virus 1 (CHV-1) infection in male dogs is manifested by lesions on the base of the penis and foreskin. There is a lack of information on the influence of these microorganisms on the quality of canine semen. Seroprevalence of *Chlamydophila* spp. (55%–61%) and CHV-1 (22%–81%) in Europe is high. The prevalence of *Chlamydophila* spp. and CHV-1 has been evaluated using polymerase chain reaction (PCR) only in Sweden and Croatia, respectively. No positive samples were detected in either case. The aim of this study was to evaluate the epidemiological situation in Polish male dogs (PMDs) to provide a solution to limit the spread of these microorganisms using assisted reproduction techniques or elimination from the reproduction of CHV-1 carriers. In addition, we assessed the semen quality of *Chlamydophila* spp. carriers and CHV-1 carriers.

**Materials and Methods:**

Cotton swabs were collected from prepuce or semen from each dog (n = 130). Real-time PCR for *Chlamydophila* spp. and CHV-1, as well as semen analysis, was performed using the computer-assisted semen analysis system.

**Results:**

To the best of our knowledge, this is the first report of *Chlamydophila* spp. infection in PMD confirmed by real-time PCR. All parameters, except progressive movement in *Chlamydophila* semen carriers, were normal.

**Conclusion:**

The average velocity values for a dog with *Chlamydia* are detailed. No CHV-1 was detected. The results achieved should be verified on the basis of a larger number of studies. However, the high prevalence of these pathogens in the PMD population has not been established.

## Introduction

The effects of *Chlamydophila* spp. and Canid herpes virus 1 (CHV-1) on male canine fertility remain unclear. In addition, there are limited data on its prevalence in canine populations. Several studies have been published on the seroprevalence of these pathogens. Studies based on polymerase chain reaction (PCR) have been performed for *Chlamydophila* spp. only in Sweden [1] and for CHV-1 in Croatia [2]. However, similar studies have not been conducted in Central Europe.

*Chlamydiaceae* are Gram-negative intracellular bacteria that attack eukaryotes. The *Chlamydiaceae* family contains 11 species and is the best-known group in the *Chlamydiales* phylum [3]. *Chlamydia* spp. and *Chlamydophila* spp. have been identified in humans, mammals (mainly koala, cattle, and cats) [4, 5], and birds [6].

However, the effects of chlamydiosis on the canine reproductive system remain poorly understood. Bitches carrying *Chlamydophila psittaci* genotype C with symptoms such as recurrent keratoconjunctivitis, respiratory disorders, and a reduction in the number of puppies in litters have been reported by Sprague *et al*. [7]. It has also been suggested that these bacteria are involved in the pathogenesis of abortion in bitches [8]. Liutkeviciene *et al*. [9] suggested that *Chlamydophila* spp. may be the main cause of canine infertility.

The pathogenesis of male reproductive tract chlamydial infection has been studied in rodents [10, 11]. These bacteria affect Leydig and Sertoli cells by dysregulating spermatogenesis and inducing apoptosis and DNA fragmentation [10]. Nielsen *et al*. [12] directly inoculated *Chlamydia trachomatis* into the prostate glands. All participants in the experiment developed benign prostatic hyperplasia.

Studies on canine chlamydiosis in Swedish kennels suggest that these infections are of little importance in terms of fertility and ocular disease since *Chlamydiaceae* bacteria have not been detected in any dog showing clinical signs [1]. However, contradictory results have also been reported by Lithuanian authors. According to published data, 61% of dogs with genitourinary signs tested positive for *Chlamydophila* spp. [9]. A case of azoospermia in a dog in which *Chlamydia* spp. was detected in the ejaculate has been described in Austria [13]. To date, reports are insufficient for clarifying the effects of *Chlamydophila* spp. on canine fertility. However, further studies are required in this field.

Previously, the diagnosis of chlamydiosis was based on cell culture, serology, and microimmunofluorescence. PCR and real-time PCR are currently the most commonly used diagnostic methods [14]. Long-term treatment of chlamydial infections (3–4 weeks) is recommended. To date, several antibiotic regimens have been developed to treat chlamydiosis, including amoxicillin with clavulanic acid (15–25 mg/kg), doxycycline (5–10 mg/kg), tetracycline (22 mg/kg), erythromycin/azithromycin (10–15 mg/kg), and enrofloxacin [15].

Herpes virus infections of the reproductive tract have been reported in dogs, cattle, and humans. CHV-1 may infect domesticated dogs and other canines, such as foxes [16]. It was first described as a cause of puppy mortality in dogs. Its prevalence in the canine population has not been estimated, but data from the literature showing seroprevalence in individual countries suggest that the virus is not sporadic [17].

CHV-1 belongs to the *Herpesviridae* family, *Alphaherpesvirinae* subfamily, and *Varicellovirus* genus [18]. Hematopoietic cells mediate the development of viremia following CHV-1 infection [16]. Similar to other herpes virus, CHV-1 can infect the respiratory and reproductive systems and cause conjunctivitis and keratitis [19]. It can cause clinical signs of upper respiratory tract infection in young animals, whereas in older animals, respiratory infection is subclinical [18].

The effects of CHV-1 on puppies are well understood. Herpes virus infection in neonates is generally fatal. Puppies may be infected during passage through the bitch’s birth canal or during contact with maternal secretions and excretions [20]. The most commonly observed signs include vocalization, anorexia, dyspnea, abdominal pain, incoordination, abnormal excrement consistency, hemorrhages, and petechiae on the mucous membrane. The mortality rate was 100% in the litter. Infections in older puppies are usually asymptomatic, although cases of neurological signs, blindness, and deafness in older puppies have been reported [21]. In addition, one case of fatal herpes virus infection in an adult dog has been reported [22]. CHV-1 infections are estimated to be the cause of death in 22.8% of infant dogs [23].

Reproductive problems caused by herpes virus infections are well understood in bitches; however, the effect of CHV-1 on male dogs is not well understood. Primary genital infections in adult bitches can manifest as vesicular lesions and congestion of the reproductive tract mucous membranes. Similar lesions at the bases of the penis and foreskin have been observed in dogs [21]. The immunization of bitches intended for breeding is recommended due to the high risk of abortion.

The effect of CHV-1 infection on the quality of canine semen has not yet been clarified; however, the reduced fertility caused by herpes virus infection in other species suggests that a similar phenomenon may occur in dogs. Bovine herpesvirus 1 (BHV1) also belongs to α-herpesviruses and is responsible for rhinotracheitis, vesicular vulvovaginal lesions, vesicular penile and preputial inflammation, and abortion and infertility [20]. BHV1 infection also negatively affects the quality of semen by decreasing the motility, viability, concentration, and percentage of morphological defects in sperm [24].

CHV-1 infection is diagnosed based on immunological (immunofluorescence, hemagglutination inhibition, and enzyme-linked immune sorbent assay) and molecular (PCR) methods [25]. To date, no method has been developed to treat herpesvirus infection. Therefore, the prevention of infection is essential. Inactivated vaccines are currently available in Europe. The vaccine should be administered to bitches during the initial stages of pregnancy and again at 6 weeks of gestation [21]. Passive immunity comes from the mother. Puppies nursed from CHV-1-seronegative bitches usually develop fatal diseases, whereas puppies from CHV-1-seropositive mothers remain asymptomatic but can still be infected [20].

The aim of this study was to estimate the prevalence of *Chlamydia* spp. and CHV-1 infection in Polish male dogs (PMD). However, there is a lack of data on the prevalence of these pathogens in Poland or their impact on the fertility of male dogs. In addition, it is necessary to clarify many discrepancies between prevalence and seroprevalence. The results of the present study could enable the future development of a protocol for use in the reproduction of *Chlamydia* spp. and CHV-1-positive dogs. This aspect appears to be of particular importance since chlamydiosis is a zoonosis. Herpesvirus is an incurable disease, and knowledge of its prevalence in dogs will help reduce its spread in the environment. The second aim was to determine whether dogs can be the primary reservoir of CHV-1, which infects bitches during mating, leading to abortion or stillbirth of puppies. The third aim was to evaluate the usefulness of PCR as a diagnostic tool for sexually transmitted diseases.

## Materials and Methods

### Ethical approval and Informed consent

The study was performed in line with the general recommendations [European Code of Good Veterinary Practice (FVE)]. Some recommendations (reduction of stress) have been adopted from the regulations (Act 15 January). All examination procedures were performed as part of the health examination at the owners’ request. According to the European Directive EU/2010/63 and Polish regulations, ethics committee approval was not required for the described procedures, which qualified as non-experimental clinical veterinary practices and were excluded from the Directive (Act of January 15, 2015, on the protection of animals used for scientific or educational purposes). All dog owners have been informed that the data obtained in this study could be used for publication.

### Study period and location

The study was conducted from March 2021 to February 2023 at Institute of Veterinary Medicine, Warsaw University of Life Sciences, Poland.

### Dogs

We enrolled 130 privately owned, intact PMDs of 61 different breeds and six mixed-breed dogs of reproductive age (1–8 years). The most common breeds were golden retriever (4), border collie (4), Bernese Mountain Retriever (3), and Nova Scotia Duck Tolling Retriever (3). Fifty-five (42.3%) of the examined dogs had previously reproduced. Body weight ranged from 1.5 kg to 80 kg, with a median (interquartile range) of 24 kg (range, 13–32 kg).

The aim of the first stage of the study was to select non-castrated dogs. Data regarding breeding history and reproductive disorders, medical history, and any medication or supplements administered over at least the previous 6 months [26] were collected for each dog. The dogs examined were not vaccinated against CHV-1 or *Chlamydophila* spp. Each dog underwent a routine clinical examination including heart and respiratory rate, rectal temperature, and lymph node palpation. Additional tests (blood count, blood biochemistry, echocardiography, or X-ray) were ordered in dogs with a questionable clinical status. These tests aimed to exclude diseases affecting semen test results (n = 12). The potential inclusion of these data does not alter the merits of this publication. Only patients with PMD without systemic diseases were included. The level of libido was evaluated using scales L1–L4, and L3 values were considered normal. Samples were obtained using cotton swabs without any transport or culture medium. Samples from the prepuce (n = 78) were obtained by rolling swabs against the mucosa or dipping swabs in the Eppendorf with semen samples (n = 52).

### Real-time PCR

Immediately after collection, the samples were stored in a refrigerator. Cotton swabs were sent to a commercial veterinary laboratory (VETLAB, Poland) on the same day to diagnose CHV-1 and *Chlamydia* spp. infection using real-time PCR.

#### Isolation of genetic material

Genetic material was isolated from cell-rich swabs using an IndiMag 48s robot (Indical, Leipzig, Germany) and dedicated DNA/RNA isolation kits (IndiMag Pathogen Kit, Indical catalog number SP947654P224), according to the manufacturer’s recommendations. The swabs were transferred to sterile Eppendorf tubes containing 400 μL of sterile 0.9% NaCl and shaken at 64× *g* for 15 min. Subsequently, 200 μL of the resulting lysate was collected and transferred to a plate using a DNA/RNA isolation kit (with the addition of proteinase K). Subsequently, 500 μL of the lysis buffer included in the kit was added.

#### Real-time PCR

Real-time PCR for *Chlamydia* spp. and CHV-1 was performed using commercial *Chlamydia*/*Chlamydophila* (Amplicon) and CHV (Amplicon) PCR kits according to the manufacturer’s instructions. For this reaction, 5 μL of isolated DNA template was obtained. The final volume of the reaction mixture was 20 μL for each reaction.

Real-time PCR conditions for *Chlamydia*/*Chlamydophila* and CHV were as follows: (1) 95°C, 5 min; (2) 95°C, 10 s; (3) 58°C, 25 s; and (4) 40°C, 30 s. Stages 2–4 were repeated in 45 cycles.

A duplicated fragment of the *Chlamydia* spp. or CHV genome was used as the positive control. With the exception of *Chlamydia*, we dealt with a putative “*Chlamydia* spp.,” which is not the exact species for positive controls. A negative control was performed on DNA-free water. The primers used in the reactions were proprietary to Amplicon sp. z o.o. (Poland) and are listed in Table-1.

**Table-1:** Sequences of primers used to perform PCR reactions.

Name	Primer sequence
Chfelis-F	CTTGCCTGTAGGGAATCC
Chfelis-R	TCTCCGTAGAATCCTGCA
CHV-F	GATTGATAGAAGAGGTATGTG
CHV-R	CCAGAATGATAAAATCCAGAA

PCR=Polymerase chain reaction, CHV-F=Canid herpes virus F, CHV-R=Canid herpes virus R

### Semen analysis

Semen was collected by manually stimulating the bulb of the penis. The level of libido was analyzed during masturbation. Three fractions were collected. Second and third fractions were evaluated. Macroscopic examination was performed at the first stage of semen evaluation. We measured and recorded the volumes of the sperm-rich fraction. The semen color and density were evaluated by the same observer. Sperm morphology was assessed by second fraction smears, followed by drying, immersion for 5 min in Sperm Stain (Microoptics, Spain), and air drying. We evaluated the samples under a light microscope (ECLIPSE E 200, NIKON, Tokyo, Japan) at 100-fold magnification. Vitality was evaluated using eosin-nigrosine staining [27]. Computer-assisted analysis was performed using a Sperm Class Analyzer (SCA version 6.5.0.67, Microoptic) combined with a Nikon Eclipse E 200 microscope (Nikon, Tokyo, Japan) and camera. The thermostable analyzer table was heated to 37°C [28]. The spermatozoa-rich fluid was diluted in phosphate-buffered saline (PBS, Sigma-Aldrich) and incubated for 5 min at 37°C before evaluation. Analyses were performed using a 20-micron GoldCyto 4 chamber slide (Goldcyto Biotechcorp, China). Curvilinear Velocity Rapid, 165 μm/s; Lin Rapid, 55%; and average head area, 20 μm^2^ were used by the manufacturer. A minimum of 500 sperm were counted and assessed for each analysis. Sperm concentration, sperm motility, round cell concentration, and mucus penetration were determined. In addition, the sperm was divided into the following subpopulations: Fast (Rapid), moderate (Medium), slow (Slow), and no movement.

### Statistical analysis

We calculated 95% confidence intervals (CI 95%) for the proportions using the Wilson score method [29].

## Results

All of the examined dogs were in good body condition with normal mentation. The dogs were not dehydrated. The rectal temperature ranged from 37.5% to 38.9% and the heart rate ranged from 80 to 100 beats/min. The respiratory rate ranged between 15 and 30 beats/min, pink and shiny mucous membranes. No abnormalities were detected in the auscultation of the heart and airways in the dogs examined. Abdominal palpation did not reveal any clinically significant symptoms. Redness in the mucus membrane of the penis or prepuce was observed in 18 dogs. Discharge in the prepuce was observed in all 14 dogs. Palpable testicles in the scrotum were detected in all dogs. No abnormalities were noted during abdominal palpation. Semen collection was performed with (45/130, 34.6%) or without females (85/130, 65.4%). The level of libido during masturbation was as follows: L1, 10 dogs; L2, 22 dogs; L3, 83 dogs; and L4, 15 dogs.

The volume of semen varies between 0.3 mL and 3 mL. The color ranged from “watered down milk” to beige. Color was correlated with ejaculate density. Clinically healthy dogs with normospermia (n = 124) or azoospermia (n = 6) were categorized.

*Chlamydia* was detected in 1/130 dogs with a prevalence of 0.8% (95% CI: 0.1%, 4.2%).

CHV was not detected in any of the 130 dogs; therefore, the prevalence was 0% (95% CI: 0%, 2.9%).

### Chlamydia positive dog

A 13-month-old Golden Retriever dog was brought to the Small Animal Clinic, Warsaw University of Life Sciences, Warsaw University of Life Sciences, Warsaw University of Life Sciences, Warsaw University of Life Sciences. Clinical examination did not reveal any pathology. Preliminary *Chlamydophila* spp. and CHV-1 swabs were collected and sent to the laboratory for analysis. The dog showed *Chlamydophila* spp.-positive results. Because the dog has not yet been used for reproduction, he cannot be sexually infected. There were no birds or dogs in the house. The most likely source of infection was wild birds used for hunting, or it could have been infected by other dogs in the kennel where it was born.

**Figure-1:**
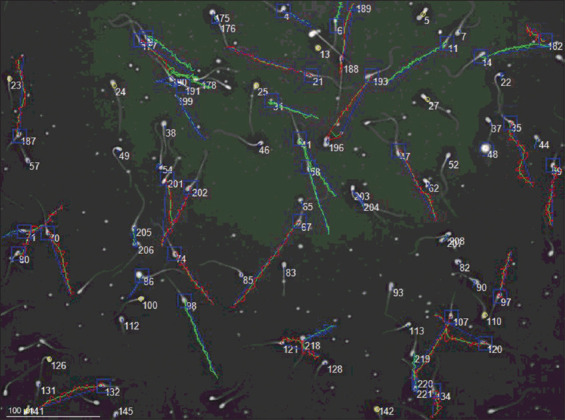
Spermatozoa of a *Chlamydia* spp.-positive dog with marked trajectories.

The semen analysis results are shown in Tables-2 [26, 30, 31] and 3. To the best of our knowledge, this is the first report of the analysis of semen from a *Chlamydophill*a spp.-positive dog using a reference method such as the computer-assisted semen analysis (CASA) system. Due to the lack of data in this area, basic semen analysis (sperm count, motility, viability, and morphology) results were compared with semen standards. The following values were considered normal: motility >70%, 200–300 million sperms per ejaculation, and morphologically normal sperm >70% [26]. All semen parameters, except progressive movement, were considered normal. Parameters such as round cells, circular tracks, mucus penetration, or the division of progressive movement into general, progressive, and moderate progressive movements, are not routinely tested in dogs. Due to this fact, a comparison with the standard could not be performed. Further research in this field is required. The trajectories of spermatozoa are shown in Figure-1.

**Table-2:** Semen parameters of *Chlamydia*-positive dog.

Semen parameter	Obtained value (%)	Reference value
Semen volume	1.9 mL	1–30 mL [30]
Concentration	145.25 million/mL	100–300 mln/mL [31]
Total concentration	275.96 million	>200–300 mln [26]
Motility	97.80	>90% [31]
Immotile	2.20	<30% [26]
General progressive motility	42.91	>70% [26]
Progressive motility	29.14	Standards are not described.
Moderate progressive motility	13.77	Standards are not described.
Non-progressive motility	54.89	Standards are not described.
Fast motility	31.94	Standards are not described.
Moderate motility	15.37	Standards are not described.
Slow motility	50.50	Standards are not described.
Round cells	0.67 million/mL	Standards are not described.
Circular tracks	56.29	Standards are not described.
Mucus penetration	43.06	Standards are not described.

**Table-3:** Average sperm velocity values for different subpopulations of *Chlamydia*-positive dog’s spermatozoa.

Average velocity value	Motile	Non-progressive motility	Moderate progressive motility	Progressive motility	Units
Curvilinear Velocity	66.93	34.04	91.47	117.28	μm/s
Average path velocity	35.67	15.13	66.17	84.26	μm/s
Linear Velocity	42.92	7.73	55	79.16	μm/s
Straightness Coefficient	38.26	35.34	84.15	93.37	%
Linearity Coefficient	59.5	16.84	62.12	67.33	%
Wobble Coefficient	52.91	37.95	72.77	71.69	%

It is unknown which species we are dealing with, some of which are zoonotic [32]. Therefore, antibiotic therapy was applied. We prescribed 10 mg/kg of doxycycline daily for 4 weeks. After treatment, the owner refused to re-examine the animal.

## Discussion

To the best of our knowledge, this is the first study to estimate the prevalence of *Chlamydophila* spp. in PMD. There is only one similar publication on the prevalence of *Chlamydophila* detected using PCR [1]. However, many studies of these pathogens in dogs have been based on serology [9]. The seroprevalence of this bacterium was estimated at 17.6% in China [33], 20% in Germany [34], and 5.5% in Slovakia [35]. This wide heterogeneity of seroprevalence makes it interesting for owners and breeders to travel all over the world with their dogs. Some authors have also suggested that the elevated titers of these bacteria may be caused by subclinical infections [36]. *Chlamydophila* spp. may inhabit parts of the body other than the reproductive system in these dogs.

*Chlamydophil*a spp. are commonly found in reptiles [4], bovines [5], birds [6] and humans [36]. Infection with *Chlamydophila* spp. in animals and humans occurs through direct contact with infected animals. Feces, urine, respiratory secretions, birth fluids, and placentas of infected animals may be sources of contamination [33]. Therefore, there is a high risk that the described case of a dog with *Chlamydophila* may have been infected while hunting birds. It should be noted that contact between dogs and other *Chlamydophila*-positive animals does not necessarily lead to infection, but may increase antibody titers against *Chlamydophila* spp.

To date, only one real-time PCR result for the prevalence of *Chlamydophila* in the canine reproductive tract has been published. Holst *et al*. [1] have estimated the presence of this bacteria in Swedish dogs. *Chlamydiaceae* were not detected in any of the 79 dogs. One *Chlamydophilla* spp.-positive sample was obtained in our study from 130 samples. In our opinion, real-time PCR is more accurate than serology because the results indicate the presence of the pathogen at the site under investigation [2]. A small amount of material is a weakness of the PCR test because the result may be false negative. In addition, in the case of a recent infection, the presence of dead pathogens which have not yet been eliminated may give false positive results. *Chlamydophila* may occur not only in the reproductive system. It has also been described in patients with coronary arteriosclerotic lesions, arthritis, pleural effusion, conjunctivitis, encephalic syndrome, bronchopneumonia, rhinitis, urethritis, and enteritis [33]. Therefore, obtaining a result with a high level of antibodies is not useful in the diagnosis of these sexually transmitted diseases because serology tests are not specific for the diagnosis of infections in the reproductive tract.

The prevalence of CHV-1 in the canine population has not yet been estimated, but data from the literature showing the seroprevalence of the virus in individual countries suggest that it occurs widely. The seroprevalence of herpesvirus in dogs with a reproduction history was 50.3% in Italy [17], 22% in South Africa [37], 39%–62.1% in Turkey [38], and 81.5% in Finland [39]. Gracin *et al*. [40] compared the seroprevalence and prevalence in dogs with Croatian infections, 32.02% of which showed positive results in the serological diagnostic methods, while all swabs tested negative for PCR.

To the best of our knowledge, Rezaei *et al*. [20] conducted the first molecular study of canine herpesvirus 1 in reproductive specimens of adult dogs in southeast Iran. In addition, Gracin *et al*.’s [40] study is based on quantitative PCR from reproductive tract swabs. Larsen *et al*. [23] performed PCR but used dead puppy tissues rather than cotton swabs. The prevalence in dead puppies was high (22.8%). There are several possible reasons why the prevalence is low in adult dogs. First, as suggested by some authors, the possibility of virus detection is very low due to brief viral excretion [40]. The virus establishes latency following viremia. PCR performed during the latent stage may result in false negative results. However, PCR has been demonstrated to be a reliable diagnostic tool even in the latent state of the virus [17]. In addition, the immune system may react quickly after contact with the virus so that infection does not occur and the antibody titers increase. In addition, CHV-1 is poorly immunogenic. Antibody concentrations may remain high for only 2 months [17]. This phenomenon suggests a high risk of false positive results. From a practical point of view, it is important to perform serological tests to decide on preventive measures, such as vaccination of pregnant bitches. Third, none of the cited studies were 3 years, and the prevalence of these viruses in the environment is dynamic. Since breeders attach great importance to bioassurance, prevention, and vaccination, the spread of the virus among breeding dogs may decrease.

## Limitation

A limitation of our study was the fact that each dog was tested only once. Therefore, the shedding phase of the virus may not have occurred in the case of CHV-1 carriers. It is well known that CHV-1 remains in the latency phase most of the time. Reactivation and shedding of CHV-1 occurs during a decline in immunity caused, for example, by stress. In future studies, we plan to test the same individual several times to capture the shedding phase of the microorganism. However, further studies are required in this field.

## Conclusion

We confirmed low prevalence of CHV-1 and *Chlamydophila* spp. in the PMD population. To the best of our knowledge, this publication is the first report of semen evaluation in the CASA system of *Chlamydophila* spp. carriers. Trajectory of sperm motility in carriers of *Chlamydophila* spp. was also described for the first time. These results should be verified on a larger number of dogs.

## Authors’ Contributions

KD and PJ: Conceptualization. KD and PJ Methodology. KD: Data analysis and writing-original draft preparation. PJ: Writing-review and editing and supervision. All the authors have read, reviewed, and approved the final manuscript.
